# Pathological Between-Network Positive Connectivity in Early Type 2 Diabetes Patients Without Cerebral Small Vessel Diseases

**DOI:** 10.3389/fnins.2019.00731

**Published:** 2019-07-16

**Authors:** Huanghui Liu, Jun Liu, Huasheng Liu, Limin Peng, Zhichao Feng, Pengfei Rong, Hui Shen, Dewen Hu, Ling-Li Zeng, Wei Wang

**Affiliations:** ^1^Department of Medical Imaging, The Third Xiangya Hospital of Central South University, Changsha, China; ^2^College of Mechatronics and Automation, National University of Defense Technology, Changsha, China

**Keywords:** type 2 diabetes, fMRI, functional connectivity, pathological between-network positive connectivity, cerebral small vessel diseases

## Abstract

**Background and Purpose:**

Previous neuroimaging studies have demonstrated type 2 diabetes (T2D)-related brain structural and functional changes are partly associated with cognitive decline. However, less is known about the underlying mechanisms. Chronic hyperglycemia and microvascular complications are the two of most important risk factors related to cognitive decline in diabetes. Cerebral small vessel diseases (CSVDs), such as those defined by lacunar infarcts, white matter hyperintensities (WMHs) and microhemorrhages, are also associated with an increased risk of cognitive decline and dementia. In this study, we examined brain magnetic resonance imaging (MRI) changes in patients in the early stages of T2D without CSVDs to focus on glucose metabolism factors and to avoid the interference of vascular risk factors on T2D-related brain damage.

**Methods:**

T2D patients with disease durations of less than 5 years and without any signs of CSVDs (*n* = 34) were compared with healthy control subjects (*n* = 24). Whole-brain region-based functional connectivity was analyzed with network-based statistics (NBS), and brain surface morphology was examined. In addition, the Montreal Cognitive Assessment (MoCA) was conducted for all subjects.

**Results:**

At the whole-brain region-based functional connectivity level, thirty-three functional connectivities were changed in T2D patients relative to those in controls, mostly manifested as pathological between-network positive connectivity and located mainly between the sensory-motor network and auditory network. Some of the connectivities were positively correlated with blood glucose level, insulin resistance, and MoCA scores in the T2D group. The network-level analysis showed between-network hyperconnectivity in T2D patients, but no significant changes in within-network connectivity. In addition, there were no significant differences in MoCA scores or brain morphology measures, including cortical thickness, surface area, mean curvature, and gray/white matter volume, between the two groups.

**Conclusion:**

The findings indicate that pathological between-network positive connectivity occurs in the early stages of T2D without CSVDs. The abnormal connectivity may indicate that the original balance of mutual antagonistic/cooperative relationships between the networks is broken, which may be a neuroimaging basis for predicting cognitive decline in early T2D patients.

## Introduction

Previous neuroimaging studies have demonstrated T2D-related brain structural (de Bresser et al., 2010; Moran et al., 2013) and functional changes (Chen et al., 2015; Wood et al., 2016), which are partly associated with cognitive decline. However, less is known about the underlying mechanisms. Cognitive decline in people with T2D is thought to progress slowly over time (Biessels et al., 2014). Brain abnormalities on MRI might serve as a proxy for long-term cognitive outcomes and the future risk of dementia. Chronic hyperglycemia and microvascular complications are two of the most important risk factors for diabetes-associated cognitive decline (Moheet et al., 2015). CSVDs, such as those defined by lacunar infarcts, WMHs and microhemorrhages, are also associated with an increased risk of cognitive decline and dementia (Brickman et al., 2012; Kitagawa et al., 2015; Akoudad et al., 2016). In this study, we tried to avoid the interference of vascular risk factors on T2D-related brain damage while focusing on glucose metabolism factors. As the T2D patients without clinical microangiopathy will inevitably have had hyperglycemic exposure, they may also show (more subtle) cerebral changes (van Duinkerken et al., 2009). Early detection of brain abnormalities at the preclinical stage can be useful for developing preventive interventions to abate cognitive decline. Thus, we explored brain structural and functional changes in the early stages of T2D patients without CSVDs.

Cognitive functioning depends on intact brain networks, which can be assessed with functional magnetic resonance imaging (fMRI) techniques. The study of resting-state functional brain networks is a powerful tool to understand the neurological bases of a variety of cognitive disorders. The pathophysiology of diabetes-related brain damage is believed to involve abnormal activity and connectivity of distributed brain networks. The majority (∼60–80%) of the resources of the brain are expended to maintain homeostasis at rest (Raichle and Mintun, 2006), suggesting that the RSNs may be particularly vulnerable in pathological conditions. Recently, a new method, NBS, has been applied in the field of whole-brain functional connectome analysis. NBS uses the graph theory concept to derive the large-scale brain connectivity with the control of familywise error (FWE) due to mass-univariate testing for each connection of the functional or structural connectome (Zalesky et al., 2010). In the current study, we sought to examine the whole-brain functional connectivity differences in the RSNs between T2D patients and healthy controls using NBS analysis to identify pathway changes in a holistic manner. FreeSurfer image analysis software based on brain surface morphology was used for brain structural analysis, and cognitive function was assessed using the MoCA. Additionally, we assessed the relationships between alterations in brain morphology or functional connectivity and the clinical variables of diabetes or MoCA scores to understand which factors were involved in brain changes. In the occurrence and development of diabetes, the order and correlation of related brain function, structure and cognitive changes are not clear. Studies have reported that changes in brain functional connectivity can be caused by structural changes, but they may also occur before clinically detectable changes in cognitive function and significant structural changes occur (Devous, 2002; Wolf et al., 2003).

We hypothesized that changes in RSNs in the early stages of T2D patients have undergone a pattern (perhaps not in most previous studies showing a simple decrease in connectivity) and that this connection change is related to the patient’s blood glucose level. At this time, the brain morphology and cognitive function of early T2D patients may not have changed. The results of this study might be helpful for understanding the neuromechanism and predicting cognitive decline in early T2D patients.

## Materials and Methods

### Subjects

In this cross-sectional study, 34 T2D patients (31–70 years, with an average age of 49.11 ± 9.68 years) and 24 age-, gender-, body mass index (BMI)-, and education-matched healthy controls (33–66 years, with an average age of 52.50 ± 9.66 years) were recruited between October 1, 2015 and February 31, 2017 from the Diabetes Outpatient Department and the Health Management Center in the Third Xiangya Hospital. All of the subjects were selected according to the following criteria: (1) all T2D patients met the diagnostic criteria proposed by the American Diabetes Association (Expert Committee on the Diagnosis, and Classification of Diabetes Mellitus, 2002) for a duration of 1–5 years before enrollment (with an average of 2.3 years); (2) there were no abnormal findings (including signs of CSVDs such as brain atrophy, lacunar infarction, encephalomalacia, cerebral microhemorrhages or WMHs) in conventional MRI sequences including T1WI, T2WI, T2-fluid attenuated inversion recovery (T2-FLAIR), and susceptibility-weighted imaging (SWI); (3) participants were 30 70 years of age; and (4) participants were right-handed. The exclusion criteria for all participants were as follows: (1) family history of dementia; (2) past history of cardiovascular or cerebrovascular diseases, neuropsychiatric diseases, brain tumor, head trauma, other chronic diseases such as thyroid dysfunction, chronic pulmonary disease, hepatic disease, or kidney disease that could influence brain function; (3) alcohol or drug abuse; (4) history of hypoglycemic episodes and insulin therapy; (5) retinopathy with a score of 1-6 indicated by fundus examination; (6) microalbuminuria, defined by an albumin-to-creatinine ratio (ACR) >2.5 mg/mmol for men and >3.5 mg/mmol for women; (7) MRI contraindications; and (8) visual, auditory and communication disorders.

The Medical Ethical Committee of the Third Xiangya Hospital of Central South University (Changsha, China) approved the study protocol in accordance with the recommendations of the Declaration of Helsinki for investigation of human participants. All participants provided written informed consent after being informed of the study details.

### Clinical Data

Each subject provided a medical history and underwent a physical examination, during which clinical data were recorded or measured with standard laboratory tests, including sex, age, education, BMI, blood pressure (BP), FPG, 2hPG, fasting insulin, fasting C-peptide, glycosylated hemoglobin A1c (HbA_1c_), blood urea nitrogen (BUN), serum creatine (SCr), total cholesterol, triglyceride (TG), high-density lipoprotein (HDL), low-density lipoprotein (LDL), urine creatinine and albumin, and fundus examination. The updated homeostasis model assessment of insulin resistance (HOMA-IR) index was calculated using the HOMA Calculator v2.2.3^1^ from FPG and fasting insulin values to evaluate insulin resistance in subjects without insulin treatment. At the same time, we obtained HOMA-β (%), i.e., the islet β-cell function index, to assess islet β-cell function.

### Cognitive Assessment

Because the subjects selected in this study were early T2D patients without CSVDs, severe cognitive impairment was not expected in these patients. Comprehensive cognitive function was assessed using the MoCA for each subject.

### Image Acquisition

All subjects underwent brain MRI examination using a 1.5-T scanner with a standard 8-channel head coil (Siemens Avanto, Erlangen, Germany). The scanning protocols were as follows: (1) the conventional brain MRI sequences included T1WI, T2WI, T2-FLAIR and SWI; (2) for resting-state function MRI (rs-fMRI), echo planar imaging (EPI) was employed with the following imaging parameters: repetition time (TR) = 2,000 ms, echo time (TE) = 40 ms, flip angle (FA) = 90°, slice thickness = 4.0 mm, slice spacing = 1.0 mm, number of slices = 28, matrix size = 128 × 128, field of view (FOV) = 240 mm × 240 mm, number of excitations (NEX) = 1.0, and scan time = 8 min 26 s; (3) high-resolution whole-brain 3D T1WI coronal scanning, applying T1-based magnetization-prepared rapid-acquisition gradient echo, was conducted with the following parameters: TR/TE = 1900/2.93 ms, FA = 15°, slice thickness = 1.0 mm, slice spacing = 0 mm, number of slices = 176, matrix size = 128 × 128, FOV = 240 mm × 240 mm, NEX = 1.0, and scan time = 7 min 3 s.

### Image Preprocessing

The raw data were transmitted to the PACS system, and images of the conventional MRI sequences were observed, excluding those images from subjects with abnormal signs such as brain atrophy, lacunar infarction, encephalomalacia, cerebral microbleeds, WMHs, or tumor.

All of the rs-fMRI data were preprocessed using previously described procedures (Zeng et al., 2014a, 2014b, 2018) with SPM (SPM8)^2^. For each subject, the first ten frames of the scanned data were discarded to avoid the magnetic saturation effect. Slice-timing and head motion correction were performed in which the remaining images were realigned to the first volume within a run for the correction of interscan head motions. All of the participants in this study had less than 2 mm translation and 2° of rotation in any of the *x*-, *y*-, and *z*-axes. Next, spatial normalization, spatial smoothing and temporal filtering were performed with the images normalized (3 mm isotropic voxels) to the standard EPI template in the Montreal Neurological Institute (MNI) space, spatially smoothed with a Gaussian filter kernel of 6 mm full-width half-maximum and temporally filtered with a Chebyshev bandpass filter (0.01–0.08 Hz). Finally, the signals that were unlikely to reflect neuronal activity were removed from the filtered images by multiple regression, including three mean signals of the white matter (WM), cerebrospinal fluid (CSF) and whole brain and six parameters obtained from head motion correction, as well as their first-order derivative terms. The residuals of the regression were used for further analysis. Based on the AAL atlas (Tzourio-Mazoyer et al., 2002), the whole brain was divided into 116 brain regions, and the signal of each region was defined by averaging the time series of all voxels within it. Subsequently, the functional connectivity between every two regions was obtained by calculating the Pearson’s correlation coefficient between the time signals of these two regions, and a 116-by-116 functional connectivity matrix was further obtained for each subject. Subsequently, Fisher’s r-to-z transformation was applied to the resulting matrix to improve normality.

The AAL atlas divided the cerebrum into 90 regions and divided the cerebellum into 26 regions. Based on this atlas, six functionally heterogeneous networks were defined by previous studies, with each network corresponding to a different functional system. Specifically, these networks included a default mode network, an attention network, a visual recognition network, an auditory network, a sensory-motor network and a subcortical network (Tao et al., 2013). In addition, the cerebellum regions were classified as cerebellum networks; therefore, the 116 regions were grouped into seven networks (Supplementary Table S1 and Supplementary Figure S1).

At the network level, this study analyzed altered within-network and between-network functional connectivity in T2D patients. We obtained two measures at the network level of analysis:

(1) The within-network connectivity strength was defined as the mean connection strength of all possible connections between each pair of the regions of interest (ROIs) in the same network for each of the seven RSNs: $W_{X}=\frac{1}{n_{X}\,\left(n_{X}-1\right)\,/\,2}\,\sum_{\mathrm{ij}\in X}z_{\mathrm{ij}}$, where *W*_*X*_ represents the mean connection strength of a specific subnetwork *X*, *n*_*X*_ represents the number of ROIs within a specific subnetwork *X* and *Z*_*ij*_ represents the functional connectivity between the ith ROI and *j*th ROI, both of which belong to the subnetwork *X*.
(2) The between-network connectivity strength was defined as the mean connection strength of all possible between-network connections between two subnetworks: $W_{X,Y}=\frac{1}{n_{X}\,n_{Y}}\,\sum_{i\in X,j\in Y}z_{i\,j}$, where *W_*X*,*y*_* represents the between-network connectivity strength of the subnetwork *X* and the subnetwork *Y*; *n*_*X*_ represents the number of ROIs within a specific subnetwork *X*; *n*_*y*_ represents the number of ROIs within a specific subnetwork *Y*; and *Z*_*ij*_ represents the functional connectivity between the *i*th ROI and *j*th ROI, one of which belongs to the subnetwork *X*, and the other to the subnetwork *Y*, where *X* and *Y* represent the subnetworks of the seven RSNs (Brier et al., 2012; Yang et al., 2016).

FreeSurfer image analysis software (vision 5.3.0)^3^ based on brain surface morphology was used for brain structural analysis (Fischl et al., 2002). Brain morphology measurements included the cortical thickness, surface area, mean curvature, gray matter volume, and subcortical region volume.

### Statistical Analyses

Demographic data, clinical variables and other measurement data were analyzed by independent sample *t*-test. The gender of the two groups was statistically analyzed by the chi-square test.

Network-based statistics analysis (Zalesky et al., 2010, 2012) was utilized to assess significant differences in the interregional connectivity matrix between the T2D and control groups. Only significant components that were significant at a FWE-corrected level of *P* < 0.05 were reported. The BrainNet viewer^4^ was used to visualize the significant components and make figures.

We used the NBS approach to identify functional connectivity that was altered in T2D patients. First, we detected the significant non-zero connections within each group by performing a non-parametric one-tailed sign test. Next, the non-zero connections within either the patient or control groups were detected and combined into a connection mask. The NBS approach was then conducted within the connection mask, where a primary threshold (*p* = 0.05) was first applied to a *t* statistic (two-sample one-tailed *t-*tests). This *t* statistic was computed for each link to define a set of suprathreshold links among which any connected components and their size (number of links) could then be determined. Then, using a non-parametric permutation approach (5,000 permutations) to estimate the significance for each component, we empirically obtained the null distribution of connected component size. For each component, we used the null distribution of maximal connected component size A to determine the corrected *P* value. To generate suprathreshold links, the same primary threshold (*t* = 3) was used, and the maximal connected component size was found. Finally, a connected component of size M between controls and patients was obtained, i.e., components showing significant changes in T2D patients in comparison to those in controls. More details of the NBS analyses can be found in Zalesky et al. (2010).

For the network-level functional connectivity analysis, to determine whether there were significant intergroup differences between the early diabetes patients and healthy controls in the resting state, two-sample *t*-tests were performed for the intranetwork connections and internetwork connections, with the significant level set at FDR (false discovery rate)- corrected *P* < 0.05.

To understand which factors were involved in the brain MRI alterations, the relationships between brain morphology alterations or changes in functional connectivity strengths and clinical variables of diabetes such as FPG, 2hPG, HbA_1c_, and HOMA-IR or MoCA scores were assessed for the T2D group, which were assessed using a general linear model simultaneously correcting the effects of age, gender, and education level, with *P* < 0.05 considered statistically significant. These assessments were performed only for the network edges that were identified in the NBS analysis as showing significant group differences. Differences in demographic and clinical variables, MoCA scores and brain morphology measures between T2D patients and controls were analyzed using two-sample *t*-test with a two-sided α-level of 0.05.

## Results

### Demographic and Clinical Characteristics

The demographic and clinical characteristics of the two groups are summarized in Table 1. There were no significant differences in age, gender, education, BMI, blood lipids, or BP between the two groups (Table 1, all *P* > 0.05, independent sample *t*-test and chi-square test). Compared with the controls, T2D patients had significantly increased FPG, 2hPG, HbA_1c_, and HOMA-IR (Table 1, all *P* < 0.05) and significantly decreased HOMA-β (Table 1, *P* < 0.05). No significant difference was found in fasting C-peptide and fasting insulin between the two groups, and there was no significant difference in the MoCA scores between the two groups (*P* = 0.058).

**TABLE 1:** Demographic and clinical characteristics of all subjects.

	**T2D group (*n* = 33)**	**Control group (*n* = 24)**	***P* value**
Age (years)	49.11±9.68	52.50±9.66	0.194
Sex (male/female, *n*)	13/20	10/14	0.970
Education (years)	13.03±3.53	11.88±3.30	0.215
BMI (kg/m^2^)	25.42±2.83	24.19±3.33	0.135
Systolic BP (mmHg)	122.78±11.51	122.92±15.66	0.969
Diastolic BP (mmHg)	77.17±11.13	75.42±7.99	0.513
Total cholesterol (mmol/l)	5.36±0.97	5.18±0.91	0.478
HDL cholesterol (mmol/l)	1.52±0.46	1.68±0.54	0.230
LDL cholesterol (mmol/l)	2.79±2.91	2.91±0.67	0.844
Cr (μmol/L)	68.21±14.82	68.79±13.46	0.878
FPG (mmol/L)	8.48±3.22	5.20±0.40	<0.001^*^
2hPG (mmol/l)	12.31±5.37	5.71±1.06	<0.001^*^
Fasting insulin (μU/ml)	12.86±9.62	10.44±6.02	0.281
Fasting C-peptide (ng/ml)	2.38±1.09	1.97±0.68	0.109
HbA_1c_ (%)	7.30±1.97	5.60±0.25	<0.001^*^
HOMA-β (%)	64.06±50.11	109.37±48.43	0.001^*^
HOMA-IR	2.02±1.30	1.25±0.76	0.012^*^
MoCA	26.32±2.42	24.38±3.99	0.058

All data are expressed as the mean ± standard deviation (SD) unless otherwise indicated. ^*^Indicates a significant difference between groups (*P* value < 0.05, independent sample *t*-test and chi-square test). T2D, type 2 diabetes mellitus; BMI, body mass index; BP, blood pressure; HDL, high-density lipoprotein; LDL, low-density lipoprotein; Cr, creatinine; FPG, fasting plasma glucose; 2hPG, 2-hour postprandial glucose; HbA_1c_, glycosylated hemoglobin A_1c_; HOMA-β, the updated homeostatic model assessment for β-cell function; HOMA-IR, the updated homeostatic model assessment for insulin resistance.

### Functional Connectivity Analysis

**FIGURE 1:**
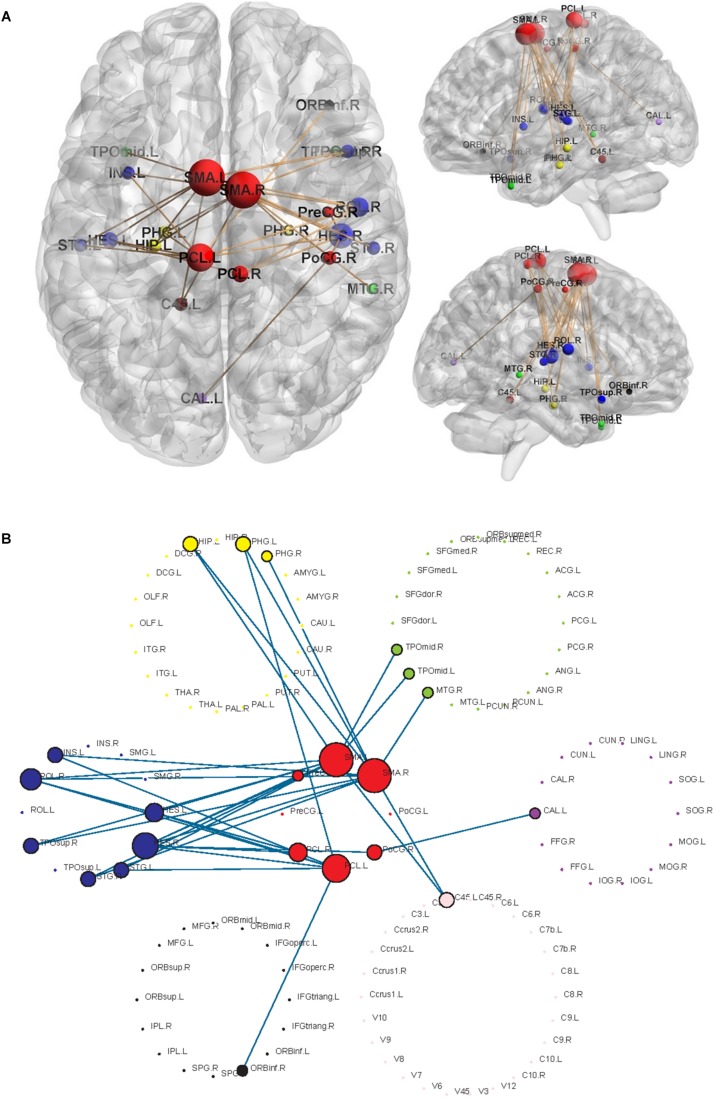
Significant networks characterizing T2D using NBS analysis. The radius of the nodes is proportional to the number of connectivities related to the nodes. Different colors represent different networks, red = sensory-motor network; blue = auditory network; yellow = subcortical network; black = attention network; green = default mode network; purple = visual network; pink = cerebellum. **(A)** The changed functional connectivities in T2D patients and the locations of the nodes involved in the brain. **(B)** Highlighted structures of 7 RSNs and changed connectivities.

**FIGURE 2:**
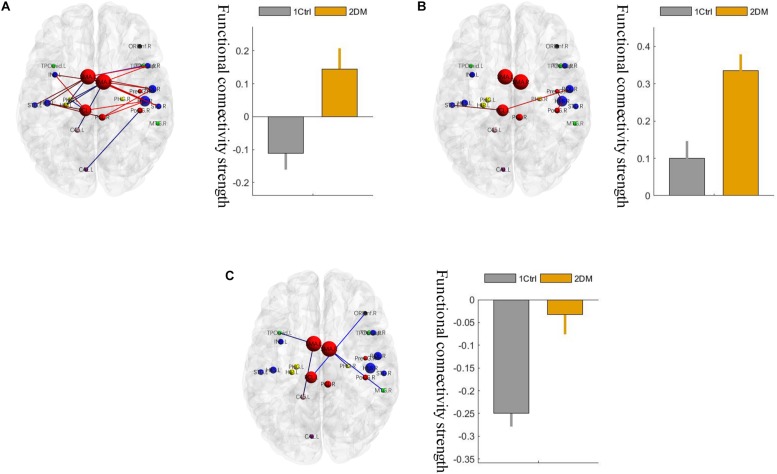
Three different changed patterns of the 33 functional connectivities. **(A)** Pathological between-network positive connectivities. Different colors represent connectivities between different networks; the red links represent the connections between the sensorimotor network and the auditory network; the blue links represent the connections that are not between the sensorimotor network and the auditory network. **(B)** Hyperconnectivity: the red links represent enhancement of positive connectivity. **(C)** Hypoconnectivity: the red links represent reduction of negative connectivity. The error bars in the figures represent the variance. 1 ctrl: the first group is the control group; 2 T2D: the second group is the type 2 diabetes group.

Using the non-parametric NBS method, we obtained a functional connectivity component with significant intergroup differences (*P* = 0.026, FWE correction) on the whole-brain connectivity level. This component consists of 33 pairs of connections and 22 brain regions (Table 2, Figures 1A,B, and Supplementary Figure S2). Twenty-four of the changes in connectivity strength in the T2D group compared to those in the control group were pathological between-network positive connectivities (here defined as a between-network positive connectivity that occurs in a disease state, which is shown as a negative connectivity in a normal state; Figure 2A), 4 were hyperconnectivity (defined as enhanced connectivity, which means that the positive connectivity value becomes larger in the current study; Figure 2B), and 5 were hypoconnectivity (defined as reduced connectivity in the current study, which means that the negative connectivity value becomes larger but is still negative; Figure 2C). These 33 functional connectivities were between-network connectivities, mostly between the sensory-motion network and auditory network, both of which are related to low-level perception. Among the regions connected with these changed connectivities, the most affected regions were the bilateral supplementary motor area and left paracentral lobule (see Supplementary Table S2 for more details).

**TABLE 2:** The 33 functional connectivities with intergroup differences and their mean connection strengths.

**ROI 1**	**ROI 2**	**T2D group**	**Control group**	***P* value**
ROL. R	SMA. L	0.1478	−0.0721	0.0045
ROL. R	SMA. R	0.1872	−0.0005	0.0057
SMA. R	INS. L	0.1095	−0.0489	0.0093
SMA. L	HIP. L	0.0639	−0.1647	0.0040
SMA. R	HIP. L	0.0644	−0.1481	0.0040
SMA. R	PHG. L	0.0006	−0.2130	0.0040
SMA. R	PHG. R	−0.0242	−0.2229	0.0041
CAL. L	PoCG. R	0.0889	−0.1174	0.0044
ORBinf. R	PCL. L	−0.0328	−0.2496	0.0033
ROL. R	PCL. L	0.1763	0.0061	0.0096
INS. L	PCL. L	0.0881	−0.0859	0.0064
PHG. L	PCL. L	0.0228	−0.1634	0.0094
ROL. R	PCL. R	0.0716	−0.1131	0.0098
SMA. L	HES. L	0.0895	−0.0758	0.0083
PCL. L	HES. L	0.2112	−0.0099	0.0004
PCL. R	HES. L	0.1180	−0.0850	0.0020
PreCG. R	HES. R	0.2606	0.0683	0.0081
SMA. L	HES. R	0.1233	−0.0942	0.0063
SMA. R	HES. R	0.1701	−0.0495	0.0024
PoCG. R	HES. R	0.3351	0.0998	0.0048
PCL. L	HES. R	0.1962	−0.0025	0.0053
PCL. R	HES. R	0.1319	−0.0627	0.0090
SMA. L	STG. L	0.1554	−0.0598	0.0094
PCL. L	STG. L	0.2069	0.0264	0.0098
SMA. L	STG. R	0.0893	−0.1603	0.0029
SMA. R	STG. R	0.1404	−0.0816	0.0032
SMA. L	TPOsup. R	0.1096	−0.1357	0.0031
SMA. R	TPOsup. R	0.1445	−0.1117	0.0034
SMA. R	MTG. R	−0.1319	−0.3170	0.0097
SMA. L	TPOmid. L	−0.0268	−0.2348	0.0089
SMA. L	TPOmid. R	0.0020	−0.1901	0.0087
SMA. R	C45. L	−0.0311	−0.2690	0.0040
SMA. R	C45. L	0.0151	−0.2065	0.0063

*CAL. L, left calcarine cortex; C45. L, left cerebellum 4 and 5; HES. L, left Heschl’s gyrus; HES. R, right Heschl’s gyrus; HIP. L, left hippocampus; INS. L, left insula; MTG. R, right middle temporal gyrus; ORBinf. R, right orbitofrontal cortex (inferior); PCL. L, left paracentral lobule; PCL. R, right paracentral lobule; PHG. L, left parahippocampal gyrus; PHG. R, right parahippocampal gyrus; PoCG. R, right postcentral gyrus; PreCG. R, right precentral gyrus; ROL. R, right rolandic operculum; SMA. L, left supplementary motor area; SMA. R, right supplementary motor area; STG. L, left superior temporal gyrus; STG. R, right superior temporal gyrus; TPOmid. L, left temporal pole (middle); TPOmid. R, right temporal pole (middle); TPOsup. R, right temporal pole (superior).*

**FIGURE 3:**
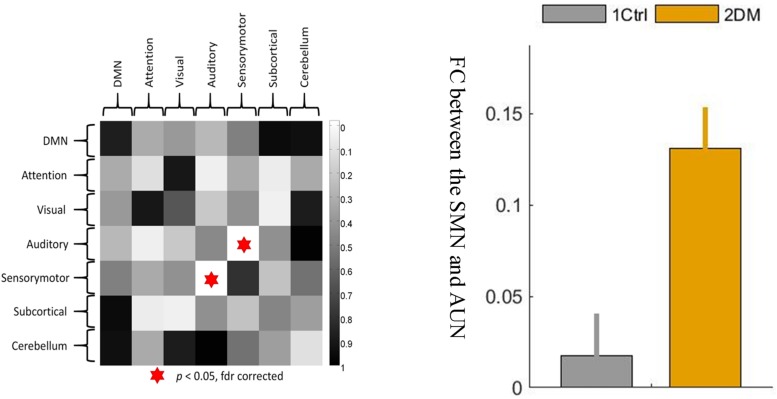
The between-network connectivity strengths between the sensory-motor network and the auditory network were significantly greater at the network level in the T2D group than in the control group. 1 ctrl: the first group is the control group; 2 DM: the second group is the type 2 diabetes group. The error bars in the figures represent the variance.

On the network level, a significant intergroup difference was only found in the connectivity strength between the sensory-motion network and the auditory network, which was significantly enhanced (hyperconnectivity) in the early T2D group (Figure 3, *p* < 0.05, FDR corrected). In addition, there were no other group differences in between-network strengths or within-network strengths.

### Brain Morphology Measurement

There were no significant differences in the brain morphology measurements, including the cortical thickness, surface area, mean curvature, gray matter volume, subcortical region volume and global gray matter, or global WM, between the two groups (all *p* > 0.05, Supplementary Tables S3–S8).

### Correlation Analysis

**FIGURE 4:**
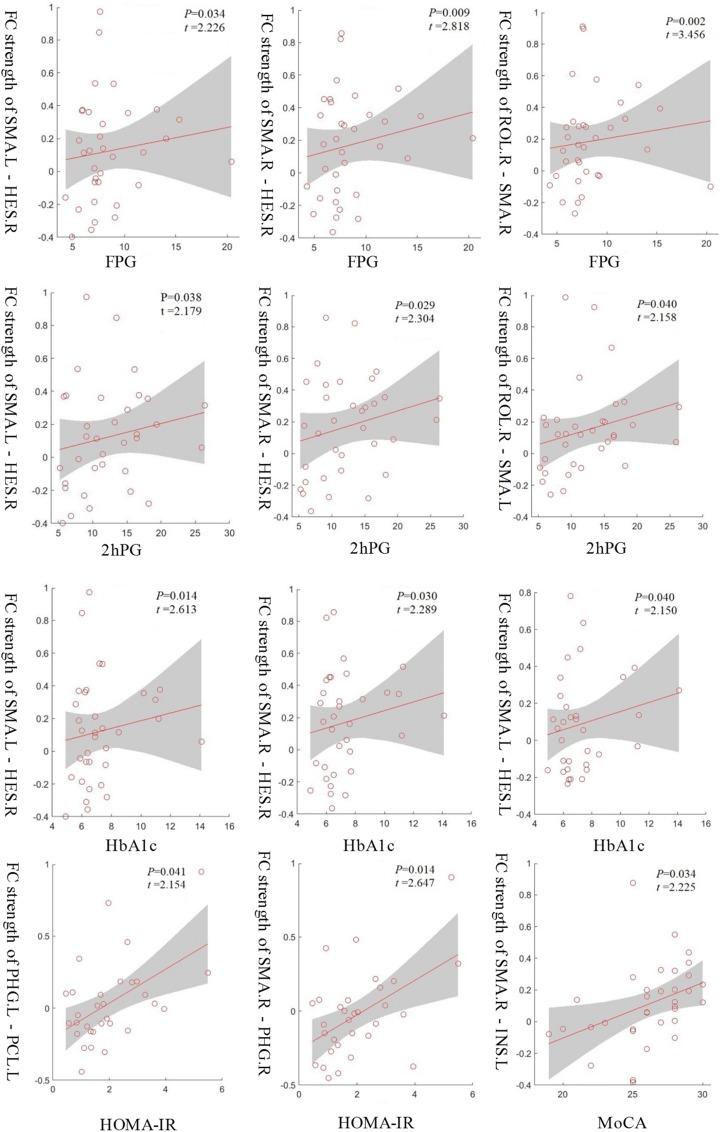
The functional connectivity strengths of the SMA.L - HES.R, SMA.R - HES.R and ROL.R - SMA.R in the T2D group were positively correlated with FPG and the strengths of the SMA.L - HES.R, SMA.R - HES.R and ROL.R - SMA.L were positively correlated with 2hPG. Significant positive correlations were also observed between the connectivity strengths of the SMA.L - HES.R, SMA.R - HES.R and SMA.L - HES.L and HbA1c and between the strengths of the PHG.L - PCL.L and SMA.R - PHG.R and HOMA-IR. Moreover, the connectivity strengths of the SMA.R - INS.L showed a significantly positive correlation with MoCA scores. *p* < 0.05, uncorrected.

To understand which factors were involved in the altered functional connectivities, we next examined the relationships between the 33 functional connectivities and clinical characteristics in the T2D group. The results demonstrated that the functional connectivity strengths of the SMA.L - HES.R, SMA.R - HES.R, and ROL.R - SMA.R were positively correlated with FPG (*p* < 0.05, uncorrected), and the strengths of SMA.L - HES.R, SMA.R - HES.R and ROL.R - SMA.L were positively correlated with 2hPG (*p* < 0.05, uncorrected). Significant positive correlations were also observed between the connectivity strengths of the SMA.L - HES.R, SMA.R - HES.R and SMA.L - HES.L and HbA1c and between the strengths of the PHG.L - PCL.L and SMA.R - PHG.R and HOMA-IR (*p* < 0.05, uncorrected). Moreover, the functional connectivity strengths of the SMA.R - INS.L in the T2D group were significantly and positively correlated with MoCA scores (*p* < 0.05, uncorrected). More details about the correlation results can be found in Figure 4. The abbreviations and full names of the brain regions mentioned above are shown in legend of Table 2.

## Discussion

In the current study, we found that compared with the control group, T2D patients without CSVDs showed altered functional connectivities that were primarily pathological between-network positive connectivities involving the connectivities between two brain regions in the sensory-motor network and auditory network related to low-level perception. Moreover, these changes in the T2D group were associated with blood glucose level, insulin resistance and MoCA scores. There was no significant difference in MoCA scores or brain morphology measurements between the two groups.

The subjects of T2D patients selected in this study were in the early stage, reflected mainly in the following three aspects. (1) The duration of diabetes is associated with cognitive impairment; that is, the longer the duration of diabetes, the more severe the cognitive impairment (Yang et al., 2016; Schneider et al., 2017). Therefore, the T2D subjects that we selected had a disease duration within 1–5 years. (2) CSVDs are also one of the risk factors for cognitive decline. Therefore, we only included subjects who had no abnormal signs on conventional brain MRI examination. (3) The current clinical methods cannot directly show the structure of cerebral small vessels, and the retinal vessels can be used as a surrogate marker of cerebral small vessels based on homology between cerebral and retinal vasculatures (Patton et al., 2005). Furthermore, microangiopathy in the eye and kidney may be used as a surrogate marker of cerebral microangiopathy, as microangiopathy is believed to be a generalized condition (Brownlee, 2005). Thus, retinal vasculopathy and microalbuminuria were included as one of the criteria for exclusion. In short, we tried to avoid the influence of vascular risk factors.

This study showed that the abnormal connections in early T2D patients in the whole brain mostly manifested as pathological between-network positive connectivity. This pattern of functional connectivity changes was also reported in a study of changes in brain function connectivity in patients with different states of consciousness (Di Perri et al., 2016). Most of the reports of brain function connectivities in the past literature showed synergistic correlations (i.e., positive connections), but anticorrelation relationships (i.e., negative connections) in brain tissue are equally important. Negative functional connectivity might come to be considered an integral part of neuronal coordination. The existence of local network anticorrelation seems to be the origin of neurons. Liu et al. (2012) reported that proper communication and coordination between two intrinsic antagonism networks was critical for information integration and maintenance of cognitive function; the resting-state abnormal connectivity of intrinsic networks may disrupt network coordination and thereby contribute to the pathophysiology of schizophrenia. Overall, the literature suggests that normal internetwork balance is needed to maintain normal cognition. The emergence of pathological between-network positive connectivities in the early stages of T2D may indicate that the original balance of mutual antagonistic/cooperative relationships between the networks is broken. Obviously, the observed changes in the current study are also a manifestation of the impaired connectivity between networks, suggesting that the original negative connections between these networks, i.e., the antagonistic relationships, are blocked and even show pathological synergistic relationships, suggesting that the neural basis of cognitive function in early T2D patients may be altered. We found that hyperconnectivity was the second pattern of functional connectivity change in early T2D patients. This hyperconnectivity change in early T2D patients may be an intermediate compensatory phase associated with functional connectivity changes (Wang et al., 2007) or a sign of functional reorganization as a compensatory response to early mild brain damage. With the prolongation of diabetes duration and increased severity of the illness, the strength of functional connectivities may decrease due to decompensation or functional reorganization failure. The third pattern of connection change we found was hypoconnectivity, which was consistent with many previous studies of brain function connectivity in diabetes (Zhou et al., 2010; Musen et al., 2012). Previous studies have confirmed that abnormal connections are associated with impaired cognitive function (Chen et al., 2014; Cui et al., 2014; Zhou et al., 2014; Zhang et al., 2015; van Bussel et al., 2016). The cognitive decline will likely accelerate or eventually develop into Alzheimer’s disease (Biessels et al., 2006; Spauwen et al., 2013).

Both at the whole-brain level and network level, all of the observed changed connectivities in the early T2D group were between networks rather than within networks, suggesting that the first damage in the early stage of T2D is damage to the antagonistic or synergistic relationship between networks and that the within-network synergy has not yet been affected. We can perhaps infer that connectivity between networks is damaged before within-network connectivity is damaged. All observed abnormal connectivities involved brain regions of the sensory-motor network and were mostly connectivities between the regions of the sensory-motor network and the regions of the auditory network, suggesting that early damage associated with T2D may be damage to low-level perception function related to the sensorimotor and auditory senses. Our results showed that the connection between the sensory-motor network and the auditory network was significantly greater in the early T2D group than in the healthy control group at the network level. van Duinkerken et al. (2012) reported that type 1 diabetes (T1D) patients without microangiopathy showed increased connectivity in networks involved in motor and visual processes, whereas T1D patients with microangiopathy showed decreased connectivity in networks involving attention, working memory, auditory and language processing, and motor and visual processes. This altered resting-state connectivity may, in part, contribute to the impairments in cognitive performance observed in T1DM patients, particularly in those with microangiopathy. This observation is similar to our results, suggesting that damaged brain regions in the early stages of T2D may be limited to neuronal input and lower-level structures involved in information processing, but this requires more research to verify.

Through correlation analysis, we found that the abnormal functional connectivity of the T2D group was positively correlated with clinical variables of diabetes, such as FPG, 2hPG, HbA_1c_, and HOMA-IR. A correlation between HbA1c levels and brain function connectivity has also been reported in previous studies (Xia et al., 2015; Liu et al., 2017). Studies have shown that poor glycemic control leading to long-term chronic exposure to hyperglycemia may be a decisive factor in T2D brain damage (Manschot et al., 2007). Insulin resistance can promote the development of mild cognitive impairment and Alzheimer’s disease (Kim et al., 2015), consistent with our findings. The observed changes in brain activity associated with T2D may be associated with disease progression, poor glycemic control, and insulin resistance. Plasma glucose levels can be controlled by regular and effective treatments, and insulin resistance can be modified by changes in lifestyle, such as diet and exercise regulation. Our results are expected to provide a basis for clinical predictive factors and new interventions. The functional connectivity strength between the SMA.R-INS.L in the T2D group and the connection strength between the sensory-motion network and the auditory network at the network level were positively correlated with MoCA score. There was no overall group difference in cognitive function between the T2D group and the healthy control group at this stage, indicating that the complex patterns of connectivity in the brain may be a putative compensatory mechanism in the early stages of T2D.

There are several limitations to this study. First, the study was a cross-sectional study with a relatively small sample size. In future studies, large-scale, longitudinal follow-up observations are needed to study the dynamic changes in brain activity and cognitive function throughout the clinical development of diabetes. Second, the neuropsychological tests used in this study are not comprehensive or sensitive, and some subtle cognitive declines may not be detected. Third, correlation analysis was not corrected for multiple comparisons. Although an association between blood glucose levels and the functional connectivity in T2D has been reported (Musen et al., 2012; Xia et al., 2013; Marder et al., 2014; Chen et al., 2015; Cui et al., 2015), we cannot exclude false-positive findings. Finally, the T2D subjects used different treatments, such as diet conditioning, exercise, and oral hypoglycemic agents; therefore, treatment-related effects may have interfered with our results.

## Conclusion

The findings of this study showed the abnormal connectivity in the early T2D patients without CSVDs, which may indicate that the original balance of mutual antagonistic/cooperative relationships between the networks is broken. Future studies based on a concept of disrupted coordination of the intrinsic networks may improve our understanding of the pathophysiological mechanisms of T2D-related brain damage.

## Ethics Statement

The Medical Ethical Committee of the Third Xiangya Hospital of the Central South University (Changsha, China) approved the study protocol in accordance with the recommendations of the declaration of Helsinki for investigation of human participants. All participants provided written informed consent after being informed of the study details.

## Author Contributions

HL and JL researched data, contributed to discussion, and wrote, reviewed, and edited the manuscript. LP and HL performed the data analysis. ZF, PR, HS, and DH contributed to the discussion and manuscript revision. L-LZ and WW made contributions to the design of the experiment and revised the manuscript. L-LZ and WW are the guarantors of this work and, as such, had full access to all the data in the study and take responsibility for the integrity of the data and the accuracy of the data analysis.

## Conflict of Interest Statement

The authors declare that the research was conducted in the absence of any commercial or financial relationships that could be construed as a potential conflict of interest.

## Footnotes

1 http://www.dtu.ox.ac.uk/homacalculator/

2 http://www.fil.ion.ucl.ac.uk/spm

3 http://surfer.nmr.mgh.harvard.edu/

4 http://www.nitrc.org/projects/bnv/

## Abbreviations

2hPG: 2-hour postprandial glucose
AAL: automated anatomical labelin
CSVDs: cerebral small vessel diseases
FPG: fasting plasma glucose
HOMA-IR: homeostasis model assessment of insulin resistance
MoCA: montreal cognitive assessment
MRI: magnetic resonance imaging
NBS: network-based statistics
rs-fMRI: resting-state function MRI
RSN: resting-state network
T2D: type 2 diabetes
WMHs: white matter hyperintensities.
