# Supplementary material for: Pathological Between-Network Positive Connectivity in Early Type 2 Diabetes Patients Without Cerebral Small Vessel Diseases
Source: Front Neurosci. 2019 Jul 16;13:731. doi: 10.3389/fnins.2019.00731 (PMC6646694; doi:10.3389/fnins.2019.00731)
Supplement: Supplementary file 1 [file Table_1.DOCX]

**Supplementary Material**

| Suppl. Table 1. The names and abbreviations of the 116 regions and 7 RSNs | | | |
| --- | --- | --- | --- |
| Regions( L/R) | Abbr. RSNs | Regions( L/R) | Abbr. RSNs |
| Superior frontal gyrus(dorsal)  Superior frontal gyrus(medial)  Orbitofrontal cortex(superior medial)  Rectus gyrus  Anterior cingulate gyrus  Posterior cingulated gyrus  Angular gyrus  Precuneus  Middle temporal gyrus  Temporal pole (middle)  Orbitofrontal cortex(superior)  Middle frontal gyrus  Orbitofrontal cortex(middle)  Inferior frontal gyrus(opercula)  Inferior frontal gyrus(triangular)  Orbitofrontal cortex (inferior)  Superior parietal gyrus  Inferior parietal lobule  Calcarine cortex  Cuneus  Lingual gyrus  Superior occipital gyrus  Middle occipital gyrus  Inferior occipital gyrus  Fusiform gyrus  Rolandic operculum  Insula  Supramarginal gyrus  Heschl gyrus  Superior temporal gyrus  Temporal pole (superior) | SFGdor. DMN  SFGmed. DMN  ORBsupmed. DMN  REC. DMN  ACG. DMN  PCG. DMN  ANG. DMN  PCUN. DMN  MTG. DMN  TPOmid. DMN  ORBsup. Attention  MFG. Attention  ORBmid. Attention  IFGoperc. Attention  IFGtriang. Attention  ORBinf. Attention  SPG. Attention  IPL. Attention  CAL. Visual  CUN. Visual  LING. Visual  SOG. Visual  MOG. Visual  IOG. Visual  FFG. Visual  ROL. Auditory  INS. Auditory  SMG. Auditory  HES. Auditory  STG. Auditory  TPOsup. Auditory | Precentral gyrus  Supplementary motor area  Postcentral gyrus  Paracentral lobule  Olfactory  Middle cingulate gyrus  Hippocampus  Parahippocampal gyrus  Amygdala  Caudate  Putamen  Pallidum  Thalamus  Inferior temporal gyrus  Cerebelum_Crus1  Cerebelum_Crus2  Cerebelum_3  Cerebelum_4_5  Cerebelum_6  Cerebelum_7b  Cerebelum_8  Cerebelum_9  Cerebelum_10  Vermis_1_2  Vermis_3  Vermis_4_5  Vermis_6  Vermis_7  Vermis_8  Vermis_9  Vermis_10 | PreCG. Sensorymotor  SMA. Sensorymotor  PoCG. Sensorymotor  PCL. Sensorymotor  OLF. SubCortical  MCG. SubCortical  HIP. SubCortical  PHG. SubCortical  AMYG. SubCortical  CAU. SubCortical  PUT. SubCortical  PAL. SubCortical  THA. SubCortical  ITG. SubCortical  Ccrus1. Cerebellum  Ccrus2. Cerebellum  C3. Cerebellum  C45. Cerebellum  C6. Cerebellum  C7b. Cerebellum  C8. Cerebellum  C9. Cerebellum  C10. Cerebellum  V12 Cerebellum  V3 Cerebellum  V45 Cerebellum  V6 Cerebellum  V7 Cerebellum  V8 Cerebellum  V9 Cerebellum  V10 Cerebellum |

| Suppl. Table 2. The networks and brain regions involved in the 33 functional connectivities with significant differences compared between the two groups   \| RSNs \| Local regions \| \| --- \| --- \| \| Sensory-motor network \| Bilateral supplementary motor area (Left: 10/66 ; Right: 10/66)  Bilateral paracentral lobule (Left: 7/66 ; Right: 3/66)  Right postcentral gyrus (2/66)  Right precentral gyrus (1/66) \| \| Auditory network \| Bilateral heschl gyrus (Left: 3/66 ; Right: 6/66)  Right rolandic operculum (4/66)  Bilateral superior temporal gyrus (Left: 2/66 ; Right: 2/66)  Right Temporal pole (superior) (2/66)  Left insula (2/66) \| \| Subcortical network \| Left hippocampus (2/66)3.80 ±0.42  Bilateral parahippocampal gyrus (Left: 2/66 ; Right: 1/66) \| \| Default mode network  Visual network  Attention network  Cerebelum \| Right middle temporal gyrus (1/66)  Bilateral temporal pole (middle) (Left: 1/66 ; Right: 1/66)  Left calcarine cortex (1/66)  Right orbitofrontal cortex (inferior) (1/66)  Left cerebellum (2/66) \| | | | | |
| --- | --- | --- | --- | --- | --- | --- | --- | --- | --- | --- | --- | --- | --- | --- |
| Suppl Table 3. Comparison of the cortical thickness between the two groups | | | |  |
| the cortical thickness(cm) | T2D Groupcm  (*n* = 34) | Control Group  (*n* = 24) | *P* value  （FWE） |  |
| Banks of the superior temporal sulcus(Left/ Right)  Cingulate cortex, Caudal anterior division(Left/ Right)  Middle frontal gyrus, Caudal division(Left/ Right)  Cuneus cortex(Left/ Right)  Entorhinal cortex(Left/ Right)  Fusiform gyrus(Left/ Right)  Inferior parietal cortex(Left/ Right)  Inferior temporal gyrus(Left/ Right)  Cingulate cortex, Isthmus division(Left/ Right)  Lateral occipital cortex(Left/ Right)  Orbitofrontal cortex, Lateral division(Left/ Right)  Lingual gyrus(Left/ Right)  Orbitofrontal cortex, Medial division(Left/ Right)  Middle temporal gyrus(Left/ Right)  Parahippocampal gyrus(Left/ Right)  Paracentral lobule(Left/ Right)  Inferior frontal gyrus, Pars opercularis(Left/ Right)  Inferior frontal gyrus, Pars orbitalis(Left/ Right)  Inferior frontal gyrus, Pars triangularis(Left/ Right)  Pericalcarine cortex(Left/ Right)  Postcentral gyrus(Left/ Right)  Posterior division(Left/ Right)  Precentral gyrus(Left/ Right)  Precuneus cortex(Left/ Right)  Cingulate cortex, Rostral anterior division(Left/ Right)  Middle frontal gyrus, Rostral division(Left/ Right)  Superior frontal gyrus(Left/ Right)  Superior parietal cortex(Left/ Right)  Superior temporal gyrus (Left/ Right)  Supramarginal gyrus(Left/ Right)  Frontal pole(Left/ Right)  Temporal pole(Left/ Right)  Transverse temporal cortex(Left/ Right)  Insula(Left/ Right) | ^2.344 ± 0.167/2.420 ± 0.167^  ^2.539 ± 0.256/2.570 ± 0.202^  ^2.475±0.173/2.503±0.164^  ^1.639 ± 0.129/1.694 ± 0.118^  ^3.482 ± 0.354/3.671 ± 0.325^  ^2.653 ± 0.142/2.627 ± 0.155^  ^2.360 ± 0.150/2.356 ± 0.127^  ^2.861 ± 0.161/2.865 ± 0.151^  ^2.381 ± 0.205/2.287 ± 0.173^  ^1.997 ± 0.119/2.092 ± 0.114^  ^2.687 ± 0.172/2.697 ± 0.176^  ^1.834 ± 0.109/1.896 ± 0.135^  ^2.582 ± 0.126/2.602 ± 0.171^  ^2.787 ± 0.183/2.849 ± 0.147^  ^2.487 ± 0.344/2.448 ± 0.233^  ^2.172 ± 0.169/2.249 ± 0.148^  ^2.545 ± 0.151/2.546 ± 0.187^  ^2.622 ± 0.233/2.630 ± 0.205^  ^2.442 ± 0.190/2.453 ± 0.128^  ^1.460 ± 0.103/1.469 ± 0.115^  ^1.957 ± 0.108/1.952 ± 0.127^  ^2.377 ± 0.154/2.409 ± 0.142^  ^2.366 ± 0.136/2.363 ± 0.136^  ^2.237 ± 0.132/2.241 ± 0.142^  ^2.847 ± 0.188/2.992 ± 0.192^  ^2.375 ± 0.138/2.350 ± 0.133^  ^2.722 ± 0.161/2.730 ± 0.152^  ^2.050 ± 0.103/2.045 ± 0.132^  ^2.619 ± 0.158/2.692 ± 0.153^  ^2.435 ± 0.134/2.425 ± 0.155^  ^2.876 ± 0.281/2.690 ± 0.273^  ^3.669 ± 0.275/3.811 ± 0.282^  ^2.144 ± 0.201/2.162 ± 0.214^  ^3.004 ± 0161/2.922 ± 0.182^ | ^2.421 ± 0.140/2.463 ± 0.169^  ^2.480 ± 0.278/2.562 ± 0.235^  ^2.539±0.131/2.494±0.192^  ^1.588 ± 0.113/1.607 ± 0.137^  ^3.422 ± 0.330/3.602 ± 0.368^  ^2.664 ± 0.105/2.645 ± 0.127^  ^2.280 ± 0.116/2.288 ± 0.138^  ^2.817 ± 0.149/2.862 ± 0.151^  ^2.376 ± 0.252/2.313 ± 0.220^  ^1.969 ± 0.126/2.064 ± 0.098^  ^2.695 ± 0.167/2.677 ± 0.157^  ^1.825 ± 0.097/1.887 ± 0.073^  ^2.600 ± 0.144/2.630 ± 0.186^  ^2.797 ± 0.144/2.821 ± 0.183^  ^2.542 ± 0.350/2.481 ± 0.235^  ^2.131 ± 0.153/2.212 ± 0.146^  ^2.608 ± 0.149/2.611 ± 0.162^  ^2.536 ± 0.230/2.702 ± 0.217^  ^2.415 ± 0.192/2.460 ± 0.173^  ^1.419 ± 0.140/1.449 ± 0.098^  ^1.983 ± 0.150/1.968 ± 0.143^  ^2.391 ± 0.181/2.403 ± 0.126^  ^2.327 ± 0.164/2.317 ± 0.194^  ^2.206 ± 0.126/2.176 ± 0.140^  ^2.849 ± 0.233/2.962 ± 0.215^  ^2.422 ± 0.174/2.433 ± 0.190^  ^2.755 ± 0.157/2.758 ± 0.158^  ^2.030 ± 0.165/1.979 ± 0.123^  ^2.647 ± 0.185/2.653 ± 0.192^  ^2.421 ± 0.140/2.426 ± 0.156^  ^2.930 ± 0.401/2.847 ± 0.349^  ^3.741 ± 0.307/3.809 ± 0.297^  ^2.108 ± 0.218/2.092 ± 0.192^  ^3.030 ± 0.135/2.955 ± 0.116^ | ^0.570/0.846^  ^0.846/0.924^  ^0.570/0.984^  ^0.643 / 0.507^  ^0.861/0.846^  ^0.861/0.846^  ^0.570/0.570^  ^0.846/0.909^  ^0.897/0.987^  ^0.846/0.846^  ^0.894/0.909^  ^0.909/0.937^  ^0.850/0.894^  ^0.850/0.909^  ^0.846/0.846^  ^0.846/0.846^  ^0.507/0.570^  ^0.846/0.643^  ^0.907/0.850^  ^0.846/0.850^  ^0.846/0.846^  ^0.924/0.909^  ^0.863/0.846^  ^0.846/0.643^  ^0.908/0.989^  ^0.643/0.507^  ^0.846/0.846^  ^0.846/0.507^  ^0.846/0.863^  ^0.924/0.924^  ^0.861/0.507^  ^0.846/0.909^  ^0.909/0. 846^  ^0.846/0.846^ |  |
|  | | | |  |

| Suppl. Table 4. Comparison of the surface area between the two groups | | | |
| --- | --- | --- | --- |
| the surface area(mm^2^) | T2D Group  (*n* = 34) | Control Group  (*n* = 24) | *P* value（FWE） |
| Banks of the superior temporal sulcus(Left/ Right)  Cingulate cortex, Caudal anterior division(Left/ Right)  Middle frontal gyrus, Caudal division(Left/ Right)  Cuneus cortex(Left/ Right)  Entorhinal cortex(Left/ Right)  Fusiform gyrus(Left/ Right)  Inferior parietal cortex(Left/ Right)  Inferior temporal gyrus(Left/ Right)  Cingulate cortex, Isthmus division(Left/ Right)  Lateral occipital cortex(Left/ Right)  Orbitofrontal cortex, Lateral division(Left/ Right)  Lingual gyrus(Left/ Right)  Orbitofrontal cortex, Medial division(Left/ Right)  Middle temporal gyrus(Left/ Right)  Parahippocampal gyrus(Left/ Right)  Paracentral lobule(Left/ Right)  Inferior frontal gyrus, Pars opercularis(Left/ Right)  Inferior frontal gyrus, Pars orbitalis(Left/ Right)  Inferior frontal gyrus, Pars triangularis(Left/ Right)  Pericalcarine cortex(Left/ Right)  Postcentral gyrus(Left/ Right)  Posterior division(Left/ Right)  Precentral gyrus(Left/ Right)  Precuneus cortex(Left/ Right)  Cingulate cortex, Rostral anterior division(Left/ Right)  Middle frontal gyrus, Rostral division(Left/ Right)  Superior frontal gyrus(Left/ Right)  Superior parietal cortex(Left/ Right)  Superior temporal gyrus (Left/ Right)  Supramarginal gyrus(Left/ Right)  Frontal pole(Left/ Right)  Temporal pole(Left/ Right)  Transverse temporal cortex(Left/ Right)  Insula(Left/ Right) | ^1043.9 ± 147.5/920.4 ± 127.7^  ^702.1 ± 183.6/818.7 ± 153.0^  ^2426.1 ± 448.3/2165.2 ± 381.6^  ^1480.1 ± 192.8/1530.0 ± 199.9^  ^428.6 ± 66.3/354.4 ± 87.5^  ^3132.4 ± 380.9/3026.9 ± 407.4^  ^4909.3 ± 742.0/5669.2 ± 882.4^  ^3455.1 ± 475.7/3183.4 ± 474.9^  ^991.0 ± 208.2/980.0 ± 218.8^  ^4871.9 ± 573.0/4682.2 ± 442.8^  ^2475.4 ± 320.9/2454.4 ± 311.3^  ^3009.2 ± 309.8/3031.7 ± 393.0^  ^1847.3 ± 262.3/1752.6 ± 213.0^  ^3047.2 ± 402.6/3302.7 ± 367.4^  ^694.4 ± 111.0/661.2 ± 95.2^  ^1348.6 ± 176.5/1586.8 ± 244.2^  ^1632.4 ± 275.4/1342.9 ± 239.5^  ^611.4 ± 74.5/738.9 ± 98.2^  ^1256.6 ± 164.6/1540.7 ± 218.8^  ^1415.2 ± 212.9/1589.1 ± 245.9^  ^4310.1 ± 635.4/4122.2 ± 575.5^  ^1140.1 ± 175.2/1167.3 ± 206.0^  ^5159.4 ± 597.2/5191.8 ± 725.8^  ^3803.9 ± 398.2/3963.0 ± 444.7^  ^845.2 ± 179.6/630.4 ± 129.2^  ^5857.9 ± 758.4/5838.3 ± 654.3^  ^7330.0 ± 831.7/7042.6 ± 906.5^  ^5582.3 ± 605.3/5386.7 ± 620.2^  ^3697.9 ± 442.7/3507.1 ± 385.8^  ^4059.2 ± 623.0/3737.2 ± 446.2^  ^198.4 ± 32.1/281.9 ± 47.0^  ^488.5 ± 52.0/436.0 ± 57.3^  ^486.8 ± 82.5/352.7 ± 65.0^  ^2301.7 ± 206.6/2472.9 ± 288.4^ | ^1075.8 ± 140.8/978.7 ± 111.8^  ^639.1 ± 125.8/820.2 ± 149.3^  ^2299.4 ± 337.9/2151.3 ± 386.9^  ^1488.3 ± 201.3/1387.9 ± 329.6^  ^409.1 ± 49.6/329.0 ± 44.0^  ^3174.0 ± 315.7/3121.3 ± 255.3^  ^4815.5 ± 526.9/5644.3 ± 462.2^  ^3431.4 ± 464.2/3160.0 ± 377.9^  ^1016.9 ± 221.4/932.3 ± 155.4^  ^4811.2±5459.0/4822.2±548.1^  ^2490.0 ± 206.7/2462.9 ± 186.1^  ^3189.6 ± 404.8/3068.9 ± 374.4^  ^1838.5 ± 220.0/1788.1 ± 182.5^  ^3200.8 ± 413.5/3505.7 ± 344.6^  ^711.8 ± 111.3/689.2 ± 78.3^  ^1327.4 ± 141.4/1577.5 ± 200.5^  ^1577.0 ± 208.0/1365.8 ± 306.3^  ^600.3 ± 66.5/739.6 ± 100.2^  ^1262.2 ± 156.7/1381.0 ± 213.4^  ^1531.0 ± 220.0/1534.5 ± 282.5^  ^4468.1 ± 664.8/4239.9 ± 450.5^  ^1152.3 ± 132.4/1188.3 ± 197.0^  ^5415.6 ± 833.3/5298.0 ± 637.0^  ^3802.2 ± 404.1/3998.5 ± 513.1^  ^838.6 ± 108.3/619.8 ± 107.5^  ^5689.7 ± 647.2/5865.0 ± 604.6^  ^7129.4 ± 628.5/6829.0 ± 525.3^  ^5121.0 ± 644.1/5369.3 ± 643.4^  ^3786.3 ± 380.8/3579.6 ± 263.0^  ^4000.8 ± 445.3/3713.7 ± 496.2^  ^207.4 ± 40.7/272.0 ± 47.6^  ^474.4 ± 43.7/424.3 ± 49.5^  ^487.3 ± 73.0/358.2 ± 64.8^  ^2210.8 ± 175.0/2365.0 ± 230.7^ | ^0.854/0.573^  ^0.854/0.905^  ^0.854/0.905^  ^0.854/0.577^  ^0.854/0.854^  ^0.854/0.574^  ^0.905/0.886^  ^0.905/0.910^  ^0.854/0.905^  ^0.950/0.493^  ^0.854/0.854^  ^0.278/0.854^  ^0.934/0.648^  ^0.379/0.278^  ^0.854/0.521^  ^0.905/0.905^  ^0.910/0.869^  ^0.910/0.905^  ^0.869/0.281^  ^0.278/0.905^  ^0.740/0.756^  ^0.854/0.854^  ^0.578/0.854^  ^0.869/0.869^  ^0.905/0.916^  ^0.854/0.869^  ^0.869/0.886^  ^0.278/0.905^  ^0.648/0.854^  ^0.998/0.952^  ^0.854/0.854^  ^0.854/0.869^  ^0.905/0.905^  ^0.577/0.756^ |
|  | | | |

| Suppl. Table 5. Comparison of the mean curvature between the two groups | | | |
| --- | --- | --- | --- |
| the mean curvature | T2D Group  (*n* = 34) | Control Group  (*n* = 24) | *P* value（FWE） |
| Banks of the superior temporal sulcus(Left/ Right)  Cingulate cortex, Caudal anterior division(Left/ Right)  Middle frontal gyrus, Caudal division(Left/ Right)  Cuneus cortex(Left/ Right)  Entorhinal cortex(Left/ Right)  Fusiform gyrus(Left/ Right)  Inferior parietal cortex(Left/ Right)  Inferior temporal gyrus(Left/ Right)  Cingulate cortex, Isthmus division(Left/ Right)  Lateral occipital cortex(Left/ Right)  Orbitofrontal cortex, Lateral division(Left/ Right)  Lingual gyrus(Left/ Right)  Orbitofrontal cortex, Medial division(Left/ Right)  Middle temporal gyrus(Left/ Right)  Parahippocampal gyrus(Left/ Right)  Paracentral lobule(Left/ Right)  Inferior frontal gyrus, Pars opercularis(Left/ Right)  Inferior frontal gyrus, Pars orbitalis(Left/ Right)  Inferior frontal gyrus, Pars triangularis(Left/ Right)  Pericalcarine cortex(Left/ Right)  Postcentral gyrus(Left/ Right)  Posterior division(Left/ Right)  Precentral gyrus(Left/ Right)  Precuneus cortex(Left/ Right)  Cingulate cortex, Rostral anterior division(Left/ Right)  Middle frontal gyrus, Rostral division(Left/ Right)  Superior frontal gyrus(Left/ Right)  Superior parietal cortex(Left/ Right)  Superior temporal gyrus (Left/ Right)  Supramarginal gyrus(Left/ Right)  Frontal pole(Left/ Right)  Temporal pole(Left/ Right)  Transverse temporal cortex(Left/ Right)  Insula(Left/ Right) | ^0.121 ± 0.015/0.121 ± 0.011^  ^0.152 ± 0.012/0.154 ± 0.010^  ^0.143 ± 0.008/0.139 ± 0.007^  ^0.171 ± 0.007/0.169 ± 0.009^  ^0.149 ± 0.018/0.146 ± 0.021^  ^0.158 ± 0.008/0.154 ± 0.010^  ^0.146 ± 0.006/0.147 ± 0.007^  ^0.154 ± 0.009/0.155 ± 0.010^  ^0.156 ± 0.008/0.154 ± 0.010^  ^0.166 ± 0.012/0.164 ± 0.008^  ^0.161 ± 0.009/0.165 ± 0.012^  ^0.167 ± 0.006/0.169 ± 0.007^  ^0.174 ± 0.014/0.162 ± 0.013^  ^0.150 ± 0.007/0.149 ± 0.010^  ^0.129 ± 0.015/0.128 ± 0.017^  ^0.148 ± 0.014/0.145 ± 0.013^  ^0.138 ± 0.009/0.138 ± 0.009^  ^0.170 ± 0.014/0.166 ± 0.010^  ^0.145 ± 0.009/0.147 ± 0.009^  ^0.170 ± 0.015/0.164 ± 0.012^  ^0.142 ± 0.007/0.141 ± 0.007^  ^0.157 ± 0.009/0.159 ± 0.009^  ^0.153 ± 0.012/0.151 ± 0.010^  ^0.150 ± 0.007/0.148 ± 0.006^  ^0.160 ± 0.012/0.160 ± 0.011^  ^0.157 ± 0.006/0.157 ± 0.005^  ^0.152 ± 0.008/0.151 ± 0.008^  ^0.143 ± 0.007/0.143 ± 0.007^  ^0.131 ± 0.008/0.129 ± 0.007^  ^0.147 ± 0.007/0.143 ± 0.007^  ^0.200 ± 0.021/0.207 ± 0.023^  ^0.163 ± 0.0170.173 ± 0.019^  ^0.145 ± 0.013/0.150 ± 0.015^  ^0.147 ± 0.009/0.163 ± 0.014^ | ^0.122 ± 0.015/0.128 ± 0.013^  ^0.150 ± 0.017/0.155 ± 0.010^  ^0.138 ± 0.010/0.137 ± 0.014^  ^0.172 ± 0.016/0.171 ± 0.011^  ^0.147 ± 0.022/0.142 ± 0.020^  ^0.156 ± 0.019/0.153 ± 0.022^  ^0.142 ± 0.008/0.145 ± 0.007^  ^0.154 ± 0.007/0.154 ± 0.013^  ^0.157 ± 0.009/0.156 ± 0.009^  ^0.162 ± 0.013/0.162 ± 0.007^  ^0.162 ± 0.009/0.163 ± 0.012^  ^0.168 ± 0.019/0.169 ± 0.011^  ^0.176 ± 0.016/0.158±0.009^  ^0.148±0.005/0.146±0.009^  ^0.128±0.019/0.125±0.011^  ^0.151±0.019/0.144 ± 0.015^  ^0.136 ± 0.009/0.139 ± 0.011^  ^0.163 ± 0.012/0.166 ± 0.014^  ^0.145 ± 0.010/0.145 ± 0.009^  ^0.171 ± 0.034/0.164 ± 0.013^  ^0.143 ± 0.009/0.143 ± 0.013^  ^0.155 ± 0.009/0.156 ± 0.010^  ^0.153 ± 0.015/0.151 ± 0.013^  ^0.147 ± 0.009/0.147 ± 0.006^  ^0.166 ± 0.019/0.158 ± 0.011^  ^0.155 ± 0.007/0.156 ± 0.010^  ^0.151 ± 0.010/0.149 ± 0.010^  ^0.141 ± 0.009/0.142 ± 0.012^  ^0.131 ± 0.010/0.131 ± 0.015^  ^0.144 ± 0.008/0.146 ± 0.011^  ^0.204 ± 0.023/0.201 ± 0.017^  ^0.173 ± 0.020/0.172 ± 0.024^  ^0.145 ± 0.016/0.147 ± 0.021^  ^0.142 ± 0.007/0.159 ± 0.013^ | ^0.928/0.504^  ^0.928/0.928^  ^0.406/0.928^  ^0. 928/0. 928^  ^0. 928/0. 928^  ^0. 928/0. 928^  ^0.406/0.638^  ^0.928/0.928^  ^0. 928/0.928^  ^0.703/0. 928^  ^0. 928/0. 928^  ^0.960/0. 928^  ^0.928/0.703^  ^0. 887/0.703^  ^0. 928/0. 928^  ^0.928/0.928^  ^0.703/0.966^  ^0.406/0.928^  ^0. 928/0. 928^  ^0. 928/0.928^  ^0. 928/0. 928^  ^0. 928/0.703^  ^0. 928/0. 928^  ^0.638/0. 928^  ^0.703/0.928^  ^0.703/0. 928^  ^0. 928/0.887^  ^0.703/0.928^  ^0. 928/0. 928^  ^0.638/0.928^  ^0. 928/0.887^  ^0.703/0. 928^  ^0.928/0.928^  ^0.406/0. 928^ |
|  | | | |

| Suppl. Table 6. Comparison of the gray matter volume between the two groups | | | |
| --- | --- | --- | --- |
| the gray matter volume (mm^3^) | T2D Group  (*n* = 34) | Control Group  (*n* = 24) | *P* value  （FWE） |
| Banks of the superior temporal sulcus(Left/ Right)  Cingulate cortex, Caudal anterior division(Left/ Right)  Middle frontal gyrus, Caudal division(Left/ Right)  Cuneus cortex(Left/ Right)  Entorhinal cortex(Left/ Right)  Fusiform gyrus(Left/ Right)  Inferior parietal cortex(Left/ Right)  Inferior temporal gyrus(Left/ Right)  Cingulate cortex, Isthmus division(Left/ Right)  Lateral occipital cortex(Left/ Right)  Orbitofrontal cortex, Lateral division(Left/ Right)  Lingual gyrus(Left/ Right)  Orbitofrontal cortex, Medial division(Left/ Right)  Middle temporal gyrus(Left/ Right)  Parahippocampal gyrus(Left/ Right)  Paracentral lobule(Left/ Right)  Inferior frontal gyrus, Pars opercularis(Left/ Right)  Inferior frontal gyrus, Pars orbitalis(Left/ Right)  Inferior frontal gyrus, Pars triangularis(Left/ Right)  Pericalcarine cortex(Left/ Right)  Postcentral gyrus(Left/ Right)  Posterior division(Left/ Right)  Precentral gyrus(Left/ Right)  Precuneus cortex(Left/ Right)  Cingulate cortex, Rostral anterior division(Left/ Right)  Middle frontal gyrus, Rostral division(Left/ Right)  Superior frontal gyrus(Left/ Right)  Superior parietal cortex(Left/ Right)  Superior temporal gyrus (Left/ Right)  Supramarginal gyrus(Left/ Right)  Frontal pole(Left/ Right)  Temporal pole(Left/ Right)  Transverse temporal cortex(Left/ Right)  Insula(Left/ Right) | ^2433.4 ± 399.9/2214.4 ± 371.3^  ^1910.4 ± 579.3/2351.9 ± 417.9^  ^6389.7 ± 1291.7/5958.2 ± 1289.7^  ^2554.1 ± 446.3/2761.9 ± 438.1^  ^2134.0 ± 243.3/1891.8 ± 440.8^  ^9807.6 ± 1496.6/9238.4 ± 1647.8^  ^12638.6 ± 2178.9/14748.1 ± 2598.8^  ^11876.5 ± 2042.7/11036.1 ± 1918.6^  ^2523.0 ± 498.4/2472.3 ± 497.5^  ^10490.9 ± 1477.8/10759.9 ± 1275.3^  ^7263.6 ± 985.4/7182.5 ± 986.4^  ^5958.8 ± 909.2/6252.7 ± 1042.3^  ^5459.6 ± 700.4/5217.3 ± 704.0^  ^10265.7 ± 1718.9/11452.2 ± 1597.0^  ^2040.4 ± 333.0/1907.9 ± 273.0^  ^3113.3 ± 580.0/3779.2 ± 639.0^  ^4705.2 ± 961.0/3819.4 ± 714.1^  ^1992.6 ± 311.5/2384.2 ± 389.2^  ^3420.8 ± 622.3/4306.5 ± 657.0^  ^1897.4 ± 362.0/2186.5 ± 400.0^  ^9260.5 ± 1712.4/8898.2 ± 1605.4^  ^2963.7 ± 469.7/3129.7 ± 603.3^  ^12886.7 ± 1916.4/12887.2 ± 1953.7^  ^9223.6 ± 1086.1/9595.9 ± 1438.5^  ^2713.5 ± 615.0/2196.1 ± 469.0^  ^15501.6 ± 2086.9/15240 ± 1949.9^  ^22287.2 ± 2948.9/21384.1 ± 3171.5^  ^12469.9 ± 1560.7/12093.9 ± 1655.3^  ^11052.4 ± 1656.5/10842.5 ± 1474.3^  ^10821.5 ± 1858.4/9849.8 ± 1392.6^  ^803.4 ± 147.5/1017.2 ± 151.5^  ^2559.5 ± 297.3/2395.8 ± 350.9^  ^1130.4 ± 210.2/851.8 ± 199.2^  ^6969.3 ± 795.1/7317.4 ± 757.4^ | ^2586.4 ± 383.4/2394.8 ± 341.9^  ^1672.3 ± 447.6/2252.0 ± 380.3^  ^6202.9 ± 928.7/5803.8 ± 1095.4^  ^2473.3 ± 404.6/2377.8 ± 626.0^  ^1975.9 ± 305.9/1757.7 ± 306.1^  ^9863.1 ± 1199.6/9530.2 ± 1071.1^  ^12051.2 ± 1454.2/14211.5 ± 1689.7^  ^11481.0 ± 1646.7/10783.1 ± 1600.6^  ^2559.1 ± 538.8/2392.6 ± 393.3^  ^10257 ± 1014.9/10920 ± 1249.0^  ^7245.5 ± 697.6/7079.9 ± 733.9^  ^6324.5 ± 1003.5/6225.2 ± 784.1^  ^5414.7 ± 594.0/5404.8 ± 483.3^  ^10795.7 ± 1448.2/11934.7 ± 1415.5^  ^2147.3 ± 304.2/2028.7 ± 312.3^  ^3014.0 ± 404.4/3708.7 ± 604.1^  ^4645.2 ± 693.3/3974.4 ± 831.0^  ^1869.6 ± 299.4/2433.1 ± 360.8^  ^3387.7 ± 520.5/3779.4 ± 675.5^  ^2005.3 ± 389.2/2078.9 ± 413.0^  ^9773.8 ± 1601.8/9096.9 ± 1071.5^  ^2970.4 ± 387.6/3135.6 ± 480.8^  ^13263.8 ± 1753.3/12875 ± 1489.5^  ^9022.5 ± 996.0/9296.5 ± 1093.0^  ^2684.8 ± 339.0/2121.7 ± 403.8^  ^15266.2 ± 1567.7/15769.9 ± 2112.3^  ^21982.2 ± 2053.1/21018.6 ± 1995.9^  ^11364.7 ± 1847.5/11567 ± 1557.0^  ^11345.5 ± 1343.6/10874.3 ± 1015.0^  ^10620.9 ± 1321.7/9802.9 ± 1190.6^  ^846.3 ± 229.4/1040.2 ± 201.1^  ^2537.3 ± 303.5/2300.3 ± 309.1^  ^1108 ± 185.3/810.7 ± 129.0^  ^6742.5 ± 675.9/7116.0 ± 616.9^ | ^0.451/0.451^  ^0.650/0.888^  ^0.955/0.999^  ^0. 951/0. 451^  ^0. 451/0. 758^  ^0. 945/0. 559^  ^0.843/0.951^  ^0.951/0.999^  ^0. 888/0.951^  ^0.951/0. 650^  ^0. 906/0. 955^  ^0.451/0. 951^  ^0.999/0.451^  ^0. 451/0.451^  ^0. 451/0.451^  ^0.888/0.999^  ^0.951/0.672^  ^0.672/0.758^  ^0. 951/0. 451^  ^0. 591/0.906^  ^0. 466/0. 758^  ^0. 888/0.951^  ^0. 591/0. 951^  ^0.955/0. 906^  ^0.945/0.999^  ^0.999/0. 451^  ^0. 951/0.955^  ^0.451/0.834^  ^0. 559/0. 939^  ^0.999/0.955^  ^0. 888/0.888^  ^0.955/0. 888^  ^0.999/0.888^  ^0.781/0. 863^ |
|  | | | |

| Suppl. Table 7. Comparison of the subcortical regions volume between the two groups | | | |
| --- | --- | --- | --- |
| the subcortical regions volume(mm^3^) | T2D Group  (*n* = 34) | Control Group  (*n* = 24) | *P* value（FWE） |
| Thalamus_Proper (Left/ Right)  Caudate (Left/ Right)  Putamen (Left/ Right)  Pallidum (Left/ Right)  Brain_Stem  Hippocampus (Left/ Right)  Amygdala (Left/ Right)  Accumbens_area (Left/ Right) | ^8142.1 ± 1012.5/7290.1 ± 849.7^  ^3582.7 ± 453.3/3429.4 ± 457.1^  ^4950.0 ± 696.4/4797.6 ± 683.4^  ^1359.8 ± 260.4/1388.8 ± 192.6^  ^21156.5 ± 1859.9^  ^3979.5 ± 526.5/4067.4 ± 401.6^  ^1404.9 ± 181.0/1439.5 ± 170.3^  ^461.6 ± 96.1/425.7 ± 83.2^ | ^8104.2 ± 743.2/7191.7 ± 705.0^  ^3334.7 ± 452.2/3233.1 ± 412.2^  ^4690.8 ± 583.9/4612.6 ± 442.1^  ^1254.3 ± 190.9/1353.9 ± 111.8^  ^21834.0 ± 2497.9^  ^4028.8 ± 429.7/4083.0 ± 406.7^  ^1431.4 ± 207.0/1495.0 ± 205.8^  ^454.7 ± 84.8/434.6 ± 82.2^ | ^0.650/0.951^  ^0.451/0.591^  ^0.758/0.888^  ^0.650/0. 951^  ^0. 451^  ^0.758/0.888^  ^0.559/0.451^  ^0.951/0.834^ |
|  | | | |

| Suppl. Table 8. Comparison of brain volume between the two groups | | | |
| --- | --- | --- | --- |
|  | T2D Group  (*n* = 34) | Control Group  (*n* = 24) | *P* value  （FWE） |
| Global gray matter(10^5^×mm^3^)  Global white matter(10^5^×mm^3^) | 4.83 ± 0.58  5.75 ± 0.57 | 4.80 ± 0.4  5.67 ± 0.44 | 0.49  0.64 |

**
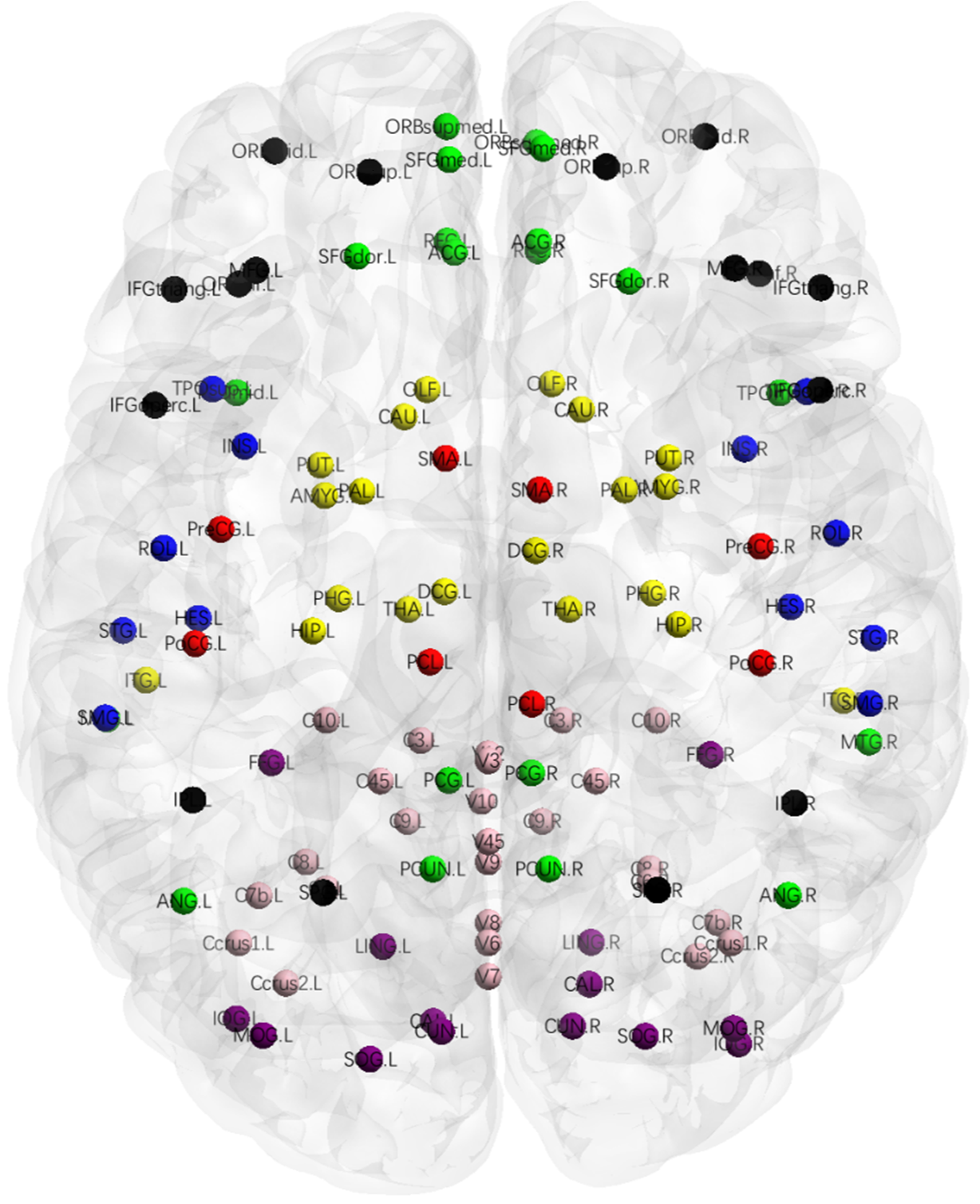
**

Suppl. Fig. 1 The distribution of 116 brain regions based on the AAL template

**
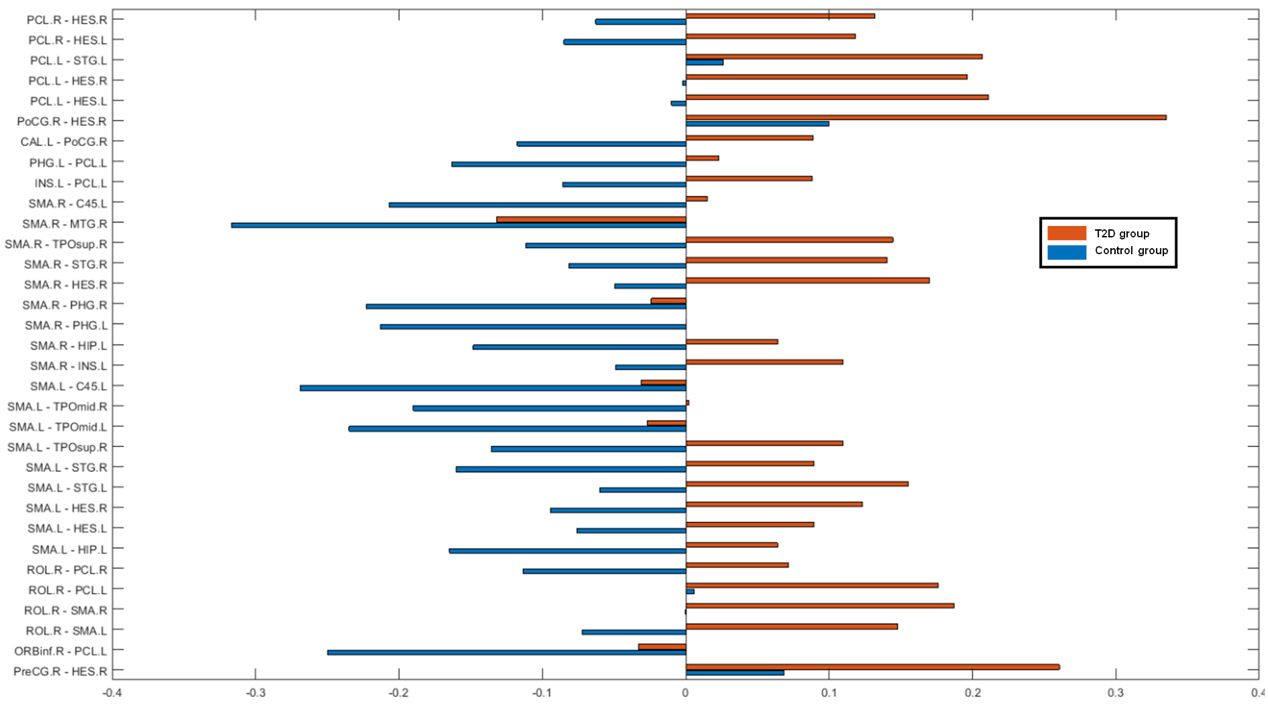
**

Suppl. Fig. 2 The 33 functional connectivities with intergroup differences and their mean connectivity strengths
